# Integrated Care Services for Dementia and Their Challenges from a Nursing Home Perspective: An Ethnographic Study

**DOI:** 10.5334/ijic.8592

**Published:** 2024-12-13

**Authors:** Taomei Zhang, Xi Wen, Yuanyuan Jin, Lu Lin, Huiling Li

**Affiliations:** 1 School of Nursing, Suzhou Medical College, Soochow University, No. 1 Shizi Street, Suzhou, CEP: 215006, China; 2 The First Affiliated Hospital of Soochow University/School of Nursing, Soochow University, No. 1 Shizi Street, Suzhou 215006, China

**Keywords:** dementia, integrated care, Rainbow model, ethnographic study, nursing homes

## Abstract

**Background:**

The number of older adults living with dementia is increasing in China and worldwide. There is limited attention paid to dementia care in nursing homes, and this study aimed to explore the current situation and challenges of providing integrated care services in nursing home settings.

**Methods:**

A 3-month focused ethnographic study, including semi-participatory observations and in-depth interviews, was conducted in a nursing home in Suzhou, China, from June to August 2022. Twelve residents and sixteen caregivers were observed, and sixteen observed caregivers were interviewed. The Rainbow Model guided data collection. Deductive analysis was used to examine the integrated care services, and a combination of deductive and inductive analysis was applied to explore the challenges.

**Results:**

The Combination of Medical and Elderly care and Medical Consortia were two main integrated care services. Eighteen challenges that threatened integrated care were identified. The nursing homes mainly faced professional and clinical challenges, while Medical Consortia encountered challenges at all levels, especially the organizational level.

**Conclusions:**

The integrated care services of residents living with dementia should be further strengthened. Policymakers, caregivers, and researchers should make more precise efforts to address the challenges that threaten integrated care services, thereby promoting better services for residents.

## Background

Dementia is a common global public health concern [1]. Alzheimer’s Disease International [2] has reported that one person is diagnosed with dementia every three seconds worldwide. As of 2020, an estimated 55 million people globally were living with dementia. The number of people with dementia is expected to reach 78 million by 2030 and 140 million by 2050 [2]. Dementia prevalence in adults aged ≥60 years in China showed that there are approximately 15.07 million older adults living with dementia [3]. The number of older adults living with dementia is increasing in China and worldwide, and dementia costs are significant both in China and worldwide [2].

In such circumstances, dementia care in nursing homes should be paid more attention to due to the following reasons. First, China’s family structure has gradually turned to the 4-2-1 model [4], which means that a young couple must provide for four older adults and one child at the same time. When children consider their own lives, take care of new family members, etc., they lack sufficient time, energy, and professional skills to provide care for older adults [5], which weakens the family’s role in providing care. The number and scale of nursing homes in China have grown rapidly and become an important support for the social service system [6]. Second, in addition to some residents (the term residents refers to older adults in nursing homes) with dementia and disabilities, many residents tend to have multiple chronic diseases such as hypertension and diabetes. The coexistence of multiple chronic diseases greatly increases the risk of cognitive disorders in residents [7,8,9]. Nursing homes tend to have more and more residents living with dementia [10]. Therefore, providing dementia care services for residents will be more effective and efficient.

Integrated care, as a service design principle, has shown promising potential in improving care quality, reducing care burden and improving care experience [11,12,13] among older adults with complex needs. However, many studies have focused on integrated care for older adults living in the community [14,15]. Furthermore, some studies used the avoided utilization of nursing homes as an important indicator to evaluate the effectiveness of integrated care [12]. Overall, the integrated care of residents living with dementia in nursing homes has sometimes been overlooked. Besides, the current literature mostly focuses on the integration at the micro level, such as case management, individual care, etc. More attention should be paid to investing certain efforts and resources at the meso and micro levels, to ensure the implementation of relevant measures [15]. China has put forward to integrate, people-centered health services and is trying to make the services more comprehensive, accessible, continuous, and coordinated [16]. Understanding the current situation and challenges of integrated care services for dementia would be beneficial for further promoting integrated care. The origins of integrated care can be traced back to the ancient Greeks, and recently it became commonplace in 1970 in long-term care for older adults [11]. Over the years, integrated care has been a widely discussed topic in healthcare management and health system design. The definition of integrated care from the World Health Organization was adopted in this study and refers to the management and delivery of health services such that people receive a continuum of health promotion, disease prevention, diagnosis, treatment, disease-management, rehabilitation and palliative care services, through the different levels and sites of care within the health system, and according to their needs throughout the life course [13]. In this study, we specifically defined the integrated care as ensuring that residents living with dementia could obtain the services they needed through the different levels and sites of care from across health and social system. Many frameworks have been developed to understand the key elements that comprise successful integrated care, such as the Chronic Care Model and its variations, the Rainbow model of integrated care (RMIC), and Patient Centred Medical Home [17,18,19]. RMIC developed by Pim P. Valentijn et al. provides a elegant and comprehensive way to understand the complexity of integrated care [11]. By placing people-focused and population-based care as the guiding principle of integration, it includes the range of different integration processes at the macro-level (system integration), meso-level (organizational and professional integration) and micro-level (clinical integration). Functional integration works alongside normative integration to ensure effective connectivity between the functioning of the integrated care system at various levels [11]. Therefore, it was chosen as the original framework to organize the current situation and challenges of integrated care [18,20]. Residents and service providers are the key stakeholders in integrated care and serve as the direct contacts with actual practice of integrated care. Therefore, this study aimed to explore the current situation and challenges of providing people-centered services from the perspectives of residents living with dementia and service providers, using RMIC as the framework.

**Research questions were:** (1) What integrated care services are provided to residents living with dementia, and how are these services perceived according to observations and service providers’ narratives? (2) What are the challenges in providing integrated care services?

## Materials and Methods

### Study Design

Ethnography is about telling credible, rigorous, and authentic stories. It gives voice to people in their local context, typically relying on verbatim quotations and a “thick” description of events [21]. This study was conducted according to principles of focused ethnography [22], as it enables exploration of a particular issue in a specific setting which means it focuses on subcultural groups having particular traits rather than the whole societies. It is a recommended method in conducting older people research [23] as it allows a deep understanding of a particular question and context and is carried out in an everyday setting. We adopted an ethnography approach to comprehensively analyze the current situation and challenges of integrated care. Data were gathered using semi-participatory observation and interviews.

### Study Setting

A nursing home in an economically thriving district of urban Suzhou, Jiangsu province, China, was selected as the study site. It adopted the care model that provided integrated services, including activities of daily life care, medical care, rehabilitation, etc. This model was referred to in China as “the Combination of medical and elderly care” [24]. Residents could obtain specialized care resources, however, the relationships between the nursing home and dementia specialist clinics were still under development, which was known as the Medical Consortia [24]. For older adults who had a need for accommodation and met the admission standards of nursing homes, they could apply to be admitted. Families of residents could visit them according to their needs. Overall, there were 26 nurses, 6 doctors, and 63 nursing aides. There were 157 residents, aged 70~104 years; 63 males (40.13%); and 127 (80.89%) with more than three kinds of chronic diseases. Forty-eight residents had dementia; 24 had Alzheimer’s disease; 8 had vascular dementia; 13 had cognitive dysfunction; 2 had Parkinson’s disease with dementia; and one had Lewy body dementia.

### Participants

A purposive sampling method was used to select service providers for observation and interview. The inclusion criteria were as follows: 1) formal caregivers such as nursing aides, doctors, nurses, and rehabilitation therapists, who were responsible for the diagnosis, treatment, care, or management of residents living with dementia; 2) with more than 1 year of work experience with dementia; 3) able to communicate well; and 4) volunteered to participate in this study. Service providers were excluded if they expressed they could not answer any of the interview questions according to their knowledge and experience. Eighteen service providers were asked to participate in the observation and interview, and two nursing aides were excluded. Finally, 16 service providers were included in the observation and interview.

A purposive sampling method was used to select residents for observation. The inclusion criteria were as follows: 1) residents living with dementia or those residents with other chronic conditions living in an environment with them; and 2) the willingness to participate in this study were obtained from their families for residents living with dementia or from residents with other chronic conditions. Twelve residents participated in this study.

Saturation, a widely accepted concept in qualitative research, refers to the point at which further data collection and analysis are unnecessary based on the data collected or analyzed thus far [25,26]. In our study, after observing 12 residents, and observing and interviewing16 caregivers, there were no new categories generated from them, so the data collection stopped.

### Theoretical Framework

RMIC included two scopes (person-focused vs. population-based), six types (system, organizational, professional, and clinical integration) and two enablers (functional and normative integration) [20,27]. The population-based scope was defined as residents living with dementia, and the person-focused scope was actually a norm and therefore was included in the normative integration. Two enablers acted as connectors across processes, facilitating collaboration among professionals, organizations, and systems to deliver integrated care, and were therefore included among the types of integration. Besides, this study was designed to focus on service level, so the observation and interview would be targeted at the organizational, professional and clinical levels as it was difficult to address system-level issues. Further, we discussed and identified the main elements from key literature that systematically reviewed the possible elements of these dimensions [28,29,30]. These dimensions and elements were used to design the observation and interview outlines, and to frame the current situation and challenges. Taking organizational functional position as an example, empirical questions were designed as: “How many organizations are related to integrated care services for dementia?”, “What are their function?”, and “How do these organizations coordinate with each other?” The questions were basically designed to be broad opening, which would prioritize the storytellers’ perspective.

### Data Collection Procedures

After obtaining informed consent from the nursing home administrators, the researcher entered nursing home as an “internship nurse” to collect data from June to August 2022.

Four head nurses were key informants who knew nursing home’s overall operation and arranged the researcher to carry out the observation. The researcher observed and interacted with the individuals in their presence. The researcher first made a descriptive observation of surrounding environment and communicated with residents and caregivers to establish trusting relationships. After obtaining informed consent from participants, formal observation was initiated. The researcher participated in daily care activities, chatting with them, taking them for a walk, etc., following service providers as they performed daily work. The researcher described the characteristics of every observation, such as location, time, date, etc., and took quick notes on the events. The data were collected through recording and photography and reflections were written within 24 hours after each observation. The researcher conducted a total of 58 observations. The observational data to be analyzed comprised nearly 30, 000 Chinese words and 35 photos.

Due to the impaired cognitive ability of the residents, they were not invited for interviews. The participants controlled the direction, content and pace of the interview, and the researcher listened and gently redirected them if their narratives deviated significantly from the aim of this study. The questions could be adjusted flexibly in their order in the interview. The interviews were transcribed verbatim within 24 hours and transcripts were returned to the interviewee for correction before data analysis. Nearly 80, 000 words were transcribed.

### Data Analysis

A deductive analysis was used to examine the integrated care services, and both deductive and inductive analysis were used to identify the challenges. Both shared the same preparation phase as follows. The researcher repeatedly listened to the recorded conversations, read field notes, transcribed interview texts, and classified the text into smaller content categories. Keywords, phrases, and sentences related to dementia care in field notes and interview texts were extracted and coded. To understand the current situation and challenges of integrated dementia care, all the data were reviewed for content and coded for correspondence with dimensions and elements in RMIC. Those challenges unfit with the main elements were used to create their categories based on the principle of inductive analysis, and mapped into the threatened dimensions of RMIC. The researcher read the material and wrote notes and headings in the text to generate initial coding. Codes with the same attributes were assigned to the same category. Then the lists of categories were grouped under higher order headings by comparing their similarities and differences. Data analysis started from choosing the subject to the completion of the research manuscript [21]. During the research period, data sorting and analysis were performed simultaneously and iteratively. Nvivo 12 Plus was used to assist in managing the data.

### Consideration of Rigor

Triangulation is a method used for trustworthiness and to enhance the validity and rigor of data analysis in qualitative research, especially in ethnography [21,31]. There are four types of triangulations proposed by Denzin [32], and the methodological and investigator triangulation were adopted in this study. First, the interview and observation methods were employed to ensure comprehensive data collection and analysis. Second, the design, data collection, analysis, findings and interpretations were carefully considered and discussed among the research team to improve the study’s transparency. Third, an external check on the preliminary findings and interpretations was performed, with the help of nursing home administrators and specialists from tertiary hospitals. They read the results and provided valuable insights to make results truly close to the reality. We also adopted the trustworthiness measures to increase the validity of analysis and results by using authentic citations without disclosing participants’ identities [33].

### Ethics Approval and Consent to Participate

This study was approved by the Research Ethics Committee of Soochow University. As outlined in the Declaration of Helsinki, the study followed appropriate ethical standards.

Before and throughout the study, the researcher posted an introduction to this study in the public activity area of nursing home to inform residents and service providers. Anyone unwilling to be observed could contact us. Then, based on the recommendations of key informants, we preliminarily determined residents and service providers to be included in the study. For residents living with dementia, during family visits, we fully informed their families of the research purpose, the form of data collection, and how to withdraw from the study. After obtaining their informed consent, we began the observation. For residents living with other chronic conditions, the informed consent were obtained from themselves after the information above were told. For service providers, we began the observation and recorded the interview after informing them of the same information and obtaining their informed consent. All participants (family members for residents living with dementia) understood that participation was voluntary, that consent could be withdrawn at any time and without their rights harmed. This manuscript did not contain identifiable information. The manuscript was also reviewed by the nursing home administrators, who agreed for it to be published.

## Results

### Participants Characteristics

The characteristics of observed and interviewed participants are presented in Tables 1 and 2.

**Table 1:** The characteristics of observed residents (n = 12).


CODE	SEX	AGE	MARITAL STATUS	EDUCATION	DIAGNOSIS	LENGTH OF STAY (MONTH)	JOB BEFORE RETIRED

G1	Female	83	Widow	Undergraduate	Dementia	2	Teacher

G2	Female	92	Widow	Junior high school	Dementia	24	Traditional Chinese Medicine Physician

G3	Female	81	Married	Primary school	Dementia, Cerebral infarction, Epilepsy, Hypertension, Type 2 DM, L1 Cone compression fracture	26	Pharmaceutical factory workers

G4	Female	83	Married	University	Dementia, Hypertension, CHD, DM	8	Coal mine technician

G5	Male	85	Married	University	CHD, coronary stent implantation, Type 2 DM, Hypertension, Cerebral infarction, Chronic bronchitis, Left knee osteoarthritis, Right knee meniscus tear, Depression	8	Coal mine technician

G6	Female	87	Married	University	Hypertension, Myocardial infarction, After cardiac stent implantation, After subtotal gastrectomy	2	Worker

G7	Male	86	Married	University	Hypertension, CHD, Lower limb vein plaque, Prostate hyperplasia, Postoperative colon cancer, Sleep disorder	2	Worker

G8	Female	86	Widow	Technical secondary school	Cerebral infarction, Hypertension, Type 2 DM, Sleep disorder, Knee joint degeneration	24	Accountant

G9	Female	77	Married	Technical secondary school	Dementia, Hypertension, Type 2 DM, Sleep disorder, Multiple rib fractures, Pyramidal fractures	60	Judge

G10	Female	89	Widow	High school	Dementia, CHD, Hypertension, Type 2 DM	48	Soldier (In art troupe)

G11	Male	86	Widow	High school	Dementia	27	Carpenter

G12	Female	74	Widow	Technical secondary school	DM, Arthritis	24	Nurse


Notes: Coronary heart disease, CHD; Diabetes mellitus, DM.

**Table 2:** The characteristics of observed and interviewed service providers (n = 16).


CODE	SEX	AGE	EDUCATION	TITLE	POSITION	WORK EXPERIENCE (YEAR)	INTERVIEW TIME (MIN)

Y1	Female	55	Undergraduate	Deputy chief physician	Doctor	5	60

Y2	Female	44	Postgraduate	Chief physician	Dementia specialist	15	60

Y3	Female	40	Postgraduate	Chief physician	Dementia specialist	10	30

N1	Female	36	Undergraduate	Nurse-in-charge	Head nurse	4	60

N2	Female	35	Undergraduate	Nurse-in-charge	Head nurse	4	50

N3	Female	33	Junior college	Nurse-in-charge	Nurse	2	20

N4	Female	33	Undergraduate	Nurse-in-charge	Head nurse	4	90

N5	Female	39	Undergraduate	Nurse-in-charge	Head nurse	2	80

N6	Female	38	Undergraduate	Nurse-in-charge	Nurse	9	25

N7	Female	34	Undergraduate	Nurse-in-charge	Nurse	2	30

K1	Female	31	Undergraduate	Primary level	Rehabilitation therapist	8	15

Z1	Male	25	Undergraduate	NA	Head nursing aide	3	15

Z2	Female	55	Junior school	NA	Head nursing aide	4	15

Z3	Male	50	Junior school	NA	Head nursing aide	4	20

Z4	Female	44	Junior college	NA	Nursing aide	4	21

Z5	Male	58	Junior school	NA	Nursing aide	4	15


Notes: NA, Not available.

### The Description of Integrated Care Services

**Figure 1:**
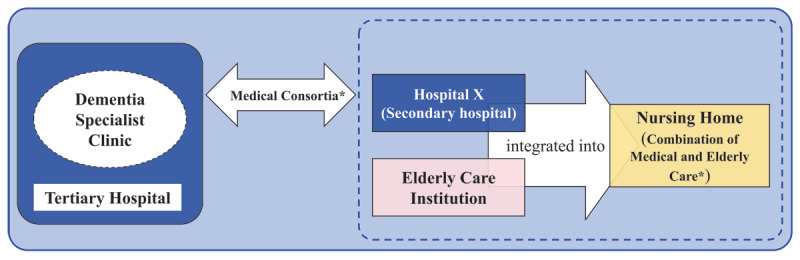
The relationships between these institutions among the integrated care services. Notes: (1)* indicates the two main integrated care services. (2) Medical Consortia: It usually integrates medical resources within the same region, typically consisting of tertiary hospitals, secondary hospitals, community hospitals and village clinics. In this study, it referred to the formal relationships between Hospital X and tertiary hospitals. (3) Combination of Medical and Elderly Care: It is usually a medical institution or elderly care institution that possess both medical and health qualifications and elderly care service capabilities. In this study, the nursing home was a Combination of Medical and Elderly Care, providing daily life care, medical treatment, nursing, rehabilitation, hospice care, psychological and spiritual support, and other services according to residents’ needs. (4) Tertiary hospital: It is a regional or higher-level hospital that provides high-level specialized medical and health services and executes higher education and research tasks to several regions. (5) Secondary hospital: It is a regional hospital that provides comprehensive medical and health services to multiple communities and undertakes certain teaching and research tasks. (6)Elderly care institution: It provides centralized residential and daily life care services for residents.

The Combination of Medical and Elderly care, and Medical Consortia were two main integrated care services. Hospital X and the elderly care institution were integrated into this nursing home, forming what is known as the Combination of Medical and Elderly Care. Additionally, Hospital X and dementia specialist clinic formed a Medical Consortia relationship. Clarification about these services and their relationships is presented in Figure 1. The implementation details at organizational, professional, and clinical levels are presented in Supplementary 1.

### The Challenges of Integrated Care Services

Eighteen challenges that threatened dementia integrated care were identified, 4 at the organizational level, 8 at the professional level, and 6 at the clinical level.

### Organizational Integration

**Challenge 1: Unformed formal relationships with dementia specialist clinics**. The nursing home relied on the Medical Consortia between Hospital X and dementia specialist clinics to receive related services. It had not yet established formal relationships with dementia specialist clinics.

Y3 said, “We formed Medical Consortia with Hospital X, but it does not mean that we need to provide services to its affiliated institutions, such as the nursing home you said. If the nursing home hopes us to provide guidance for them, we need to redraw the agreement with them.” (Observation, Y3)

“Residents living with cognitive impairment here can visit the outpatient of Hospital X and see specialists from tertiary hospitals, conveniently and timely, just within 500 meters. However, we did not directly form Medical Consortia with tertiary hospitals right now.” (Interview, N1)

**Challenge 2: No substantial benefits-sharing mechanism**. An agreement were usually signed between two parties, with their responsibilities and obligations described. The nursing home could provide people-centered services and improve their service capabilities through the guidance of specialist clinics from tertiary hospitals, and nursing home would transfer severe patients, who were unable to serve by the nursing home, to tertiary hospitals. However, tertiary hospitals were more driven by their public welfare and social responsibility to provide guidance. The lack of substantial benefits made many existing Medical Consortia a mere formality.

*“*We established Medical Consortia relationships between our tertiary hospitals and elderly care institutions, like some nursing homes. We discussed what we would do for each other. However, in most of the Medical Consortia, the medical actions we took were spontaneous and voluntary, and we did not receive corresponding financial support.” (Interview, Y2)

We learned from the formed Medical Consortia, and gained insights that the lack of substantial benefits-sharing mechanism would threaten the Medical Consortia between nursing homes and tertiary hospitals. (Reflections from interview)

**Challenge 3: Incoordination of resources**. Residents could visit dementia specialists from tertiary hospitals, both online and offline. However, the resources, such as medical technology, drugs, medical equipment, and manpower, had not been used coordinately, which greatly reduced the function of Medical Consortia and also did not align with the people-centred approach.

“I do not think the overall effect of Medical Consortia was good now. Although I was here to help residents nearby for several times, I did not make much difference. There was no team support to conduct scale measurements, no PET-CT, and no access to prescribe first-line treatment drugs.” (Interview, Y3)

**Challenge 4: Goals stuck in the agreement**. Although the goals and values of Medical Consortia were described in the agreement, the lack of benefits-sharing mechanism between both parties made the goals and values of Medical Consortia difficult to maintain and finally turned them into empty slogans.

“I think organization integration such as Medical Consortia, is a good medical guarantee for residents. It can connect medical and social service systems, allowing residents to receive more comprehensive services. However, if we do not truly establish benefits-sharing mechanism, these goals will only stay on the agreement.” (Interview, Y2)

### Professional Integration

**Challenge 1: Incomplete team structure**. Non-pharmacological interventions were very important for maintaining residents’ cognitive function and quality of life, which mainly relied on nurses and nursing aides to conduct after they finished their work. Non-pharmacological interventions should be planned and organized by social workers systematically. However, this position was vacant, hindering the accessibility and continuity of non-pharmacological interventions.

“Every 150 residents should be equipped with one social worker, but there is no such position.” (Interview, Z1)

One doctor was responsible for managing nearly 150 residents, one nurse was responsible for the health, daily care, etc., of nearly 15 residents, and one nursing aide was responsible for the daily care of 6 residents, assisting with their functional activities, such as eating, toileting, walking, etc. It was difficult to rely solely on them for the design and implementation of non-pharmacological interventions. (Observation)

**Challenge 2: Shortage of human resources**. The low social status associated with working in nursing homes and complex symptoms of dementia led to a shortage of human resources, such as nursing aides, nurses, doctors, which seriously affected the provision of people-centered services.

“Doctors and rehabilitation therapists are difficult to recruit because they have a low social status in the nursing home context. Now they even look down on secondary hospitals, let alone nursing homes.” (Interview, N1)

“Caring for residents living with dementia requires a strong care system. One nursing aide is needed to watch one resident with dementia, which is time-consuming and labor-intensive.” (Interview, N1)

**Challenge 3: Low level of knowledge**. Whether professionals with medical backgrounds or nursing aides with low education levels, they had certain ability deficiencies in dementia care services, such as non-pharmacological interventions, care skills, etc.

“Our nurses lack professional guidance. We are like crossing the river by feeling for stones. We do not have any special skills in taking care of residents living with dementia.” (Interview, N4)

Only G11 received training in the rehabilitation room. The training received by residents with dementia was the same as that received by residents with stroke. When I asked what rehabilitation training was available and specific for cognitively impaired residents, K1 said she didn’t know. (Observation, K1)

**Challenge 4: Low quality of nursing aides**. Nursing aides, who were the primary caregivers for residents living with dementia, lacked skills in daily care and managing behavioral and psychiatric symptoms of dementia. In addition, their low education level and insufficient ability to receive knowledge made improving their abilities very challenging.

“These aunts and uncles (nursing aides) have different cultural backgrounds and qualities. You can train them and tell them something, but it is very difficult for them to learn.” (Interview, N2)

**Challenge 5: Continuous impact of COVID-19**. Nursing home was often in a semi-closed state because of COVID-19. Related psychological care activities could only be dependent on professionals in nursing home. Volunteers were unable to enter nursing home for caring activities due to occasional outbreaks of the epidemic.

“In recent years, there have not been many social organizations for residents. To be honest, there are none.” (Interview, N2)

**Challenge 6: Lack of active participation among dementia specialists**. Regular public lectures and consultations were potential way of improving ability of professionals from nursing homes, however, the loss of performance evaluation and assessment made knowledge sharing between them lack sustainability and stability.

“Experts from the Neurology department of the tertiary hospitals came to our nursing home to give free lectures and consultations, but this only happened occasionally.” (Interview, N1)

**Challenge 7: No standards and norms of non-pharmacological interventions**. Non-pharmacological interventions were limited to dementia programs and usually conducted by researchers. Standards and norms of non-pharmacological interventions were lacking, mainly reflected in service contents and evaluation mechanisms. This made it difficult to apply non-pharmacological interventions to clinical practice, as staff were unable to perceive their effects and gradually lost interest in implementing them.

“It is better to have someone who can carry out non-pharmacological interventions, like social workers. And non-pharmacological interventions should be implemented and evaluated, otherwise, we do not know the significance of this work.” (Interview, N5)

**Challenge 8: Lack of performance evaluation**. Although Medical Consortia were formed in response to national calls, policies, etc., when put into practice level, professionals had to devote time and labor. The work done by dementia specialists was often of a public welfare nature, which meant their performances were not assessed, evaluated, or linked to their salary and professional title promotion. This was not conducive to enhancing their enthusiasm and making the support and guidance to nursing homes difficult to maintain.

“Our hospital established Medical Consortia with other nursing homes, but our doctors always provided services voluntarily. Therefore, we were unwilling to do this work after a period of time.” (Interview, Y2)

### Clinical Integration

**Challenge 1: Lack of a coherent process**. A relatively single or coherent process for residents living with dementia had not been formed, such as case management and individual multidisciplinary care plans, which made it impossible to create and share individual care plans among care teams and family members.

“We recorded personalized information for residents before, such as their past experiences, lifestyle habits, and other characteristics, but later on we felt it was not very useful. Over time, no one was willing to do it anymore.” (Interview, N6)

**Challenge 2: Uncertain interventions after comprehensive geriatric assessment**. The comprehensive geriatric assessment was conducted according to the evaluation criteria for older-adult-friendly hospitals, which also included the evaluation of cognitive function. However, the standards and norms for further interventions were not clear at the clinical level, which made the comprehensive geriatric assessment gradually stop.

“The publicity of comprehensive geriatric assessment was not enough, and what should we do after the assessment was not clear.” (Interview, Y1)

**Challenge 3: Temporary caregivers**. The high mobility of nursing aides and the temporary nature of dementia specialists made comprehensive and individualized care plans based on residents’ conditions (socioeconomic status, past experience, etc.) largely interrupted. These highly threatened continuous care.

A temporary caregiver was transferred from another ward. G2’s speech words were difficult to understand, and this caregiver couldn’t understand G2’s needs, so G2 wasn’t very happy today. (Observation, Y1 and G2)

**Challenge 4: Irreplaceable affection function of families**. The relationships between residents and their families were indivisible. It may be largely due to the severe impairment of their recent memory, and residual long-term memory made their mental world only contain past people and events, such as their youth and family. This kind of need was difficult to transfer or replace with activities or the companionship of caregivers, which often made caregivers feel there was nothing they could do.

“We are the people who accompany residents, but they prefer to be accompanied by their children, family members, and other blood relatives, who cannot be replaced by anything.” (Interview, N2)

G11 dozed off in a wheelchair most of the time. He was willing to open his mouth only when he was told that his daughter had prepared his meal. (Observation, G11)

**Challenge 5: Forced reduction in social interaction**. Residents living with dementia may experience behavioral and psychiatric symptoms, such as delusions, hitting, etc., which may cause them to be prejudiced and discriminated against by residents with other chronic conditions, and force their social interaction to decrease.

When I saw G12 and G8 chatting in their rooms, I went with G1 and hoped to join them. However, as soon as we entered the room, they stopped talking. Then G12 turned to me and whispered to me, “Do not bring her here. She is out of her mind.” Then they scattered. I felt G12’s discrimination and exclusion against her, and it also affected other residents. (Observation, G1, G8, and G12)

**Challenge 6: Inactive participation of family members**. When residents experienced cognitive impairment, they lost full civil autonomy. However, inactive participation of family members in decision-making, care planning, companionship, etc., hindered residents from getting personalized and proactive care, and other medical and health services.

“I told her son that his mother might have cognitive dysfunction. He refused to admit this even if the symptoms were obvious. Therefore, she did not have drugs for dementia because these drugs were psychotropic with some side effects, which needed to be signed by family members.” (Interview, N5)

## Discussion

This study explored the current situation of integrated care services and the associated challenges perceived by local stakeholders in the nursing home, focusing on residents living with dementia and service providers. Guided by RMIC, a detailed description of integrated care services was provided and 18 challenges were identified.

### Enriching the Application Of RMIC

In our study, RMIC facilitated a logical and theoretical exploration of integrated care services for residents living with dementia. RMIC was used to summarize the evidence, clinical provision and progress towards integrated primary care and social services for older adults with multimorbidity in a previous study [34], and the evidence were refined into micro, meso and macro level. In Beaudin’s study, only clinical integration was applied to older adults with chronic diseases according to the research aim [35]. It could be seen that RMIC had been used flexibly and also had strong theoretical applicability, and our study further enriched it in its scope of application in dementia care services in nursing home settings.

### Drawing on Chinese Wisdom to Advocate and Improve Integrated Dementia Care Services

The two main integrated care services were received by residents living with dementia in nursing homes in China. Combination of Medical and Elderly care could provide activities of daily life care, rehabilitation, medical care of common chronic disease etc., and Medical Consortia could help residents obtain specialized medical services, from the collaboration of various institutions and professionals. How these institutions and professionals worked with each other were described in a detailed manner, which could draw on Chinese wisdom to provide people-centered services for residents living with dementia in nursing homes. Prior research has shown uncertainty regarding the effectiveness of integrated care in nursing homes and other institutions [36,37], which may threaten the idea of advocacy for integrated care in nursing homes. In this study, accessibility, comprehensiveness, coordination, and continuity of services, and meeting residents’ needs were used as criteria to determine the challenges. Although we did not have quantitative data to accurately measure the effectiveness of integrated care, the gaps between these criteria and reality were identified as challenges and could be used to improve the services. Therefore, by addressing how integrated care services are delivered and identifying associated challenges, our findings offer valuable insights for researchers, managers, and policymakers aiming to enhance integrated dementia care services in nursing homes and ultimately improve the experiences of residents.

### Providing Multilevel Targets to Improve Services

This study identified challenges that threatened dementia integrated care across organizational, professional, and clinical perspectives, totally 18 challenges: 4 at the organizational level, 8 at the professional level, and 6 at the clinical level. A scoping review revealed that integrated care for older adults with multiple chronic diseases typically addresses only the clinical level, with only 35% of studies considering micro, meso, and macro levels [34,35]. However, successful examples of integrated care in nursing homes indicate that comprehensive integration across all levels can mitigate fragmentation risks and prevent service users from ‘falling between the cracks’ of care [12]. The challenges identified across these three levels underscore the practical significance of promoting dementia integrated care.

### Developing Strategies from the Professional and Clinical Levels to Improve Combination of Medical and Elderly Care

It mainly faced challenges from professional and clinical integration instead of organizational integration. In the context of this study, the nursing home represented a tightly integrated system originating from a secondary hospital and elderly care institutions, and formed an integrated management system. Therefore, there were no organizational challenges from them. However, issues such as the structure, quantity, and quality of professionals, as well as external environmental changes, posed threats to professional integration. Additionally, the lack of a coherent process, the irreplaceable role of family affection and forced reductions in social interaction, threatened clinical integration. First, the foremost challenge in professional integration was the shortage of social workers. Social workers play a crucial role in enhancing the continuity and coordination of interventions for older adults living with dementia through the use of case management [38]. Across different countries, the minimum standards of healthcare professions for collaborative dementia care in primary care settings involved social workers [39]. Addressing this shortfall is pivotal for improving practice. Second, there was a shortage of professionals both in terms of quality and quantity. The lower social status and salaries are pervasive issues observed in the field. Government bodies should leverage social media to shape public opinion, advocating societal support for older adults and their caregivers, thereby enhancing the social standing of elderly care professionals. Measures to bolster professional stability should include improvements in salaries and social insurance policies [40,41]. Finally, the indispensable role of family affection among residents echoes findings from previous studies [42]. In traditional Chinese culture, values such as filial piety and family reunion hold deep significance, driving a strong desire among older adults to reunite with their families. Therefore, caregivers and managers could engage family members in care by emphasizing family participation, instituting relevant policies, and strengthening research on family participation interventions.

### Establishing a Benefits-Sharing Mechanism was Crucial for the Success of Medical Consortia

Medical Consortia, a coordination between tertiary hospitals and nursing homes, were under construction and faced challenges across three levels, particularly at the organizational level, such as unformed formal relationships with dementia specialist clinics, no substantial benefits-sharing mechanism, and goals stuck in the agreement. China has made tremendous efforts with Medical Consortia construction through national-level policies and pilot projects in various regions. The first Medical Consortia in China was signed and launched in the Luwan District of Shanghai in 2011, and the National Health Commission publicly released the Management Measures for Medical Consortia in 2020 to promote its development [16]. However, this study found that despite a decade of efforts, the development of Medical Consortia in China has not been optimistic. The primary concern that threatened its effectiveness was the absence of a benefits-sharing mechanism at the organizational level. Benefits extend across the organizational, professional, and clinical levels, serving as the driving force behind the collaboration among various stakeholders. The absence of a benefits-sharing mechanism at the organizational level will directly lead to difficulties in resource sharing among institutions (such as coordinating dementia treatment drugs), professionals’ lack of performance evaluations and unwillingness to participate, and finally affected the service provision. Previous experience has also shown that improving integrated care within existing systems is highly challenging and requires substantial time for continuous adaptation and transformation [34]. Therefore, it takes time to achieve genuine and beneficial coordination between nursing homes and other organizations. Policymakers and researchers should intensify discussions on establishing a substantive benefits-sharing mechanism, while the government should strengthen supervision and assessment of the organizations involved.

## Limitations

This study had several limitations. Integrated care services can vary significantly in design and provision due to factors such as service recipients, service settings, and the specific contexts of health and social service systems in different countries [13]. Therefore, when applying the findings to design strategies in specific settings, their applicability needs careful consideration. Besides, residents with dementia were unable to fully articulate their views and needs, so their unmet needs were identified through interviews with their service providers and researcher observations, which may potentially limit a comprehensive exploration of residents’ service needs. Lastly, family members, as primary decision-makers of residents, were not included in this study. This was mainly because the nursing home was in a semi-closed state and strictly managed during this study, and family members’ visiting time was very limited. Therefore, future studies should address this gap to capture the perspectives of family members.

## Conclusions

This 3-month ethnographic study conducted in a nursing home in Suzhou contributes to the current literature by providing insights into integrated care services for residents living with dementia and the associated challenges. The identification of eighteen challenges offers valuable insights for enhancing services in nursing home settings for residents living with dementia. This study has significant implications for dementia care, the findings of which can inform researchers, policymakers, and caregivers in promoting policy formulation and optimizing the development of integrated care in long-term care facilities, thus improving the quality of life and care experiences of residents living with dementia.

## Additional File

The additional file for this article can be found as follows:

10.5334/ijic.8592.s1 Supplementary 1. Description and Challenges of Integrated Care Services.
