# Supplementary material for: Integrated Care Services for Dementia and Their Challenges from a Nursing Home Perspective: An Ethnographic Study
Source: Int J Integr Care. 2024 Dec 13;24(4):12. doi: 10.5334/ijic.8592 (PMC11639704; doi:10.5334/ijic.8592)
Supplement: Supplementary 1. — Description and Challenges of Integrated Care Services. [file ijic-24-4-8592-s1.pdf]

Supplementary 1. Description and Challenges of Integrated Care Services

Dimensions and related elements	Description of integrated care services	Challenges identified through deductive analysis	Challenges identified through inductive analysis
Organizational integration: The nursing home should provide comprehensive care to residents, such as health promotion, disease prevention, diagnosis, treatment, rehabilitation, and long-term care, by coordinating with health and social institutions. The organizational integration sub-category included elements that threatened or influenced integrated care, mainly including five parts: System structure and organizational functional position, Governance structure and collaborative relationships, Benefit and responsibility sharing, Resource construction and sharing, and Shared vision and values.			
1. System structure and organizational functional position	(1) The nursing home, which was the integration of secondary Hospital X and elderly care institutions, called Combination of Medical and Elderly care, played a crucial role in delivering comprehensive dementia services to residents. [X]<br>(2) Medical Consortia between nursing home and tertiary hospitals were under construction. Residents in this nursing home could go to Hospital X, a secondary hospital, to obtain specialist services. The formal relationship was formed between Hospital X and a tertiary hospital. [Y]	NA	NA
2.Governance structure and collaborative relationship	(1) The Governance structure and collaborative relationship between the nursing home and Hospital X was tight. The nursing home was managed by Hospital X. [X]	C1: Unformed formal relationships with dementia specialist clinics. [Y]	
3. Benefit and responsibility sharing	(1) The nursing home took responsibility for providing daily life care, long-term care, etc. for residents, and Hospital X took responsibility for medical care. Both of them could gain benefits from the payment from residents and government.[X]	C2: No substantial benefits-sharing mechanism. [Y]	
4. Resources construction and sharing	The resources construction and sharing between organizations could be mainly reflected in information, medical technology, drugs, medical equipment, manpower, etc.<br>(1) These resources were shared between the nursing home and Hospital X. [X]<br>(2) Between the nursing home and tertiary hospitals, information on disease conditions,	C3: Incoordination of resources. [Y]	

	treatment plans, etc. of residents were shared on regional information network platforms, and related examination results were mutually recognized, while not for drugs. [Y]		
5. Shared vision and values	(1) The nursing home was managed by Hospital X, so they shared same vision and values. [X]<br>(2) The organizations participating in Medical Consortia clarified the goal and value in the agreement, and related service providers gradually recognized the importance of integrated care. [Y]	C4: Goals stuck in the agreement. [Y]	
Professional integration: The professionals involved in dementia integrated care services of the nursing home, mainly including doctors, nurses, nursing aides, rehabilitation therapists, as well as dementia specialists. The professional integration sub-category included elements that threatened or influenced integrated care, respectively: Professional division and collaboration, Resource sharing, Incentive and constraint mechanisms, and Multiple professional service standards and norms.			
1. Professional division and collaboration	(1) The professionals of the nursing home formed a care team responsible for residents' daily care, basic medical care and long-term care, including doctors, nurses, nursing aides, rehabilitation therapists. Doctors were usually responsible for taking care of the common chronic diseases, such as hypertension, diabetes. Nurses were usually responsible for providing the medical care according to medical prescriptions, such as intravenous infusion. Nursing aides were usually responsible for providing daily life care, such as feeding, bathing for residents. Rehabilitation therapists were usually responsible for providing rehabilitation for residents.[X]<br>(2) Dementia specialists outside the nursing home were responsible for diagnosing and treating residents with dementia or with suspected dementia. [Y]<br>(3) Dementia specialists kept a loose relationship with professionals in the nursing home. [Y]	C1: Incomplete team structure.[X]<br>C6: Lack of active participation among dementia specialists.[Y]	C2: Shortage of human resources.[X]<br>C3: Low level of knowledge.[X]<br>C4: Low quality of nursing aides.[X]<br>C5: Continuous impact of COVID-19.[X,Y]
2. Multiple professional service norms	There were relatively clear service standards and norms for dementia prevention, diagnosis, drugs treatment and long-term care, such as guidelines and standards. [X]	C7: No standards and norms of non-pharmacological interventions.[X]	

3. Incentive and constraint mechanisms	(1) The work of professional from the nursing home were evaluated and related to their salary and bonus. [X]<br>(2) Dementia specialists usually needed to spend a certain amount of time and efforts providing guidance to service providers in the nursing home, and developing treatment and care plans, etc. for residents. Dementia specialists usually did this work voluntarily.[Y]	C8: Lack of performance evaluation.[Y]	
Clinical integration: The coordination of person-focused care in a single process across time, place and discipline. The clinical integration sub-category included elements that threatened or influenced integrated care, respectively: A single or coherent process and Residents' needs.			
1. A single or coherent process	(1) Doctors, nurses, nursing aides, etc. within the nursing home treated and cared for residents based on shared case medical records and treatment plans. The fixed service providers could contact with residents frequently, could gradually understand their past experiences, lifestyle habits, personal preferences.[X]<br>(2) Dementia specialist only came for consultations when there were changes in residents' conditions that could not be handled by doctors in the nursing home, and they would review the patient's medical history and recent situation to update treatment plans. [Y]	C1: Lack of a coherent process. [X,Y]<br>C2: Uncertain interventions after comprehensive geriatric assessment.[X]	C3: Temporary caregivers. [X,Y]<br>C5: Forced reduction in social interaction.[X]<br>C6: Inactive participation of family members.[X,Y]
2. Residents' needs	The idea of integrated care required not only caring for residents' psychological needs, but also considering their psychological and social needs. Residents became gradually dependent on the recognition and help of the outside world to meet their physiological needs with worsening of cognitive ability. Residents also had strong psychological needs, such as looking forward to their family's arrival, being eager to be cared for, accompanied, and loved by their families. In addition to physiological and psychological needs, residents with cognitive impairment also had related social communication needs. Residents also needed a friendly social interaction environment to keep normal social relationships. [X]	C4: Irreplaceable affection function of families.[X]	

Notes: "NA" indicated there were no corresponding challenges; C, Challenges; **[X]** indicated the situation and challenges were from the Combination of Medical and Elderly care (within nursing home); **[Y]** indicated the situation and potential challenges were from Medical Consortia.
